# Network meta-analysis of traditional Chinese exercises for cardiovascular health in middle-aged and older adults hypertensive patients

**DOI:** 10.3389/fspor.2026.1736747

**Published:** 2026-02-24

**Authors:** Shaolin Zhou, Zhao Zhang

**Affiliations:** 1 College of Physical Education, Qinghai Minzu University, Xining, Qinghai, China; 2 Physical Education School, Shaanxi Normal University, Xi'an, Shaanxi, China

**Keywords:** cardiovascular metabolism, hypertension, middle-aged and older adults, network meta-analysis, traditional Chinese exercises

## Abstract

**Introduction:**

Hypertension is a major public health concern, prevalent among aging populations. We aimed to compare the cardiovascular metabolic effects of four traditional Chinese exercises (Tai Chi, Baduanjin, Wuqinxi, and Yijinjing) in middle-aged and older adults hypertensive patients through network meta-analysis, and provide exercise recommendations for this population.

**Methods:**

Randomized controlled trials of traditional Chinese exercises on cardiovascular health in middle-aged and older adults hypertensive patients were systematically searched in China National Knowledge Infrastructure (CNKI), Wanfang data, Cqvip, Web of Science, PubMed, and Cochrane Library databases from January 2010 to July 2025. 19 studies with 1,501 individuals were selected and analyzed with RevMan 5.3 and Stata 15.1. The primary outcomes were blood pressure measures, lipid profiles (total cholesterol, triglycerides, high-density lipoprotein cholesterol, low-density lipoprotein cholesterol), and endothelial function indicators (nitric oxide, endothelin). Surface Under the Cumulative Ranking Area (SUCRA) probability rankings were determined to select best interventions.

**Results:**

The SUCRA probability ranking results show that in terms of improving the blood pressure of middle-aged and elderly hypertensive patients, Tai Chi demonstrated the highest probability of being the best intervention for reducing systolic and diastolic blood pressure, with SUCRA values of 86.7% and 79.3% respectively probability (63.6%).

**Discussion:**

Tai Chi and BaduanJin demonstrated a relatively high probability of superiority in improving multiple cardiovascular and metabolic indicators of middle-aged and older adults patients with hypertension. These findings support the practical application of traditional Chinese exercise regimens, Tai Chi and Baduanjin, in cardiovascular disease care strategies for middle-aged and older adults hypertensive patients.

**Systematic Review Registration:**

https://www.crd.york.ac.uk/PROSPERO/, PROSPERO CRD420251141539.

## 1 Introduction

Hypertension is a major public health concern, particularly prevalent among aging populations. According to the World Health Organization's 2023 *Global Report on Hypertension*, the number of adults with hypertension doubled from 650 million in 1990 to 1.3 billion in 2019 (1). The *China Cardiovascular Health and Disease Report* (2024) indicates that the prevalence of hypertension among Chinese adults aged ≥18 years has reached 31.6%, with higher rates in males (36.8%) than in females (26.3%) and in rural (33.7%) compared to urban (29.1%) areas, showing a marked increase with advancing age (2). This substantial global rise is driven not only by population growth and aging but also by sedentary lifestyles, unhealthy dietary habits, and rapid urbanization. This demographic shift, marked by population aging and a growing prevalence of hypertension, presents major challenges to healthcare systems worldwide, especially as 41.3% of hypertensive individuals also exhibit dyslipidemia (3).

Beyond the epidemiological burden, hypertension is intrinsically connected to endothelial dysfunction, which is characterized by increased endothelial reactivity and pathological remodeling (4). This link has been demonstrated in several global cohort studies, showing endothelial dysfunction as both a consequence and contributing factor to the etiology of hypertension (5). Exercise therapy constitutes an effective intervention for endothelial dysfunction in patients with hypertension, primarily by increasing the availability of nitric oxide (6).

Dyslipidemia exhibits a strong epidemiological association with hypertension, as evidenced by the heightened prevalence thereof among individuals with lipid abnormalities (7). Early intervention strategies that target both metabolic dysfunction and vascular health are essential because these conditions deteriorate over time, particularly in middle-aged and older adults patients.

As hypertension and related metabolic disorders become increasingly prevalent, global healthcare systems have begun to recognize the therapeutic benefits of structured exercise programs, especially those that include conventional physical activities. In China, traditional medicine hospitals have established preventive healthcare departments that incorporate traditional methods into their clinical treatment protocols. According to the *Guidelines for the Construction and Management of Preventive Healthcare Departments in Traditional Chinese Medicine Hospitals (Revised 2025 Edition)*, traditional exercise training programs such as Baduanjin and Tai Chi are essential service offerings. These guidelines emphasize the development of customized health management programs for diverse populations, encompassing individuals with prehypertension and those with stable chronic illnesses (8). This evidence-based approach is consistent with current American Heart Association recommendations for physical activity as the first-line treatment in mild-to-moderate risk hypertensive populations (9), demonstrating the growing recognition of the therapeutic benefits of structured physical activity in cardiovascular risk management.

Traditional Chinese activities may have a positive impact on certain cardiovascular risk factors. Meta-analytic evidence from Baduanjin studies shows significant benefits in various lipid markers, including lower triglyceride (TG) and total cholesterol (TC) levels, as well as higher beneficial high-density lipoprotein (HDL) values (10). However, data for low-density lipoprotein (LDL) reduction is inconsistent, with current trials indicating no meaningful effects. The extent and consistency of these lipid-modulating effects appear to vary among traditional exercise methods, with some providing stronger evidence for improved endothelial function and lipid metabolism (11–14). Furthermore, a recent systematic review and meta-analysis focusing on exercise training for cardiovascular health demonstrated the metabolic benefits of exercise interventions in detail (15).

According to traditional Chinese medicine, these exercises regulate the dynamic balance of yin and yang to harmonize visceral systems, meridians, qi circulation, and blood flow. Appropriate exercise improves qi and blood circulation throughout the body, increases liver qi flow, and boosts spleen function (16). Despite growing interest in traditional Chinese activities for hypertension management, there is a significant vacuum in the comparative data base.

While investigations on the therapeutic effects of specific traditional Chinese exercise interventions are prevalent, thorough comparison studies evaluating many traditional exercises at the same time are rare. We, therefore, aimed to utilize network meta-analysis, a well-established statistical framework for assessing numerous therapies at the same time (17), to systematically evaluate and rank different traditional Chinese exercises for improving cardiovascular metabolic outcomes in middle-aged and older adults hypertensive patients, providing essential theoretical evidence for evidence-based clinical applications.

## 2 Materials and methods

### 2.1 Literature search strategy

#### 2.1.1 Database and retrieval time

This article adheres to the Preferred Reporting Items for Systematic Reviews and Meta-Analyses (PRISMA) guidelines to conduct a meta-analysis and complete protocol registration (registration number: CRD420251141539) on the PROSPERO platform, aiming to enhance research transparency and objectivity (18). To identify relevant studies, systematic searches were executed in key Chinese and English databases, namely the China National Knowledge Infrastructure (CNKI), Wanfang data, Cqvip, Web of Science, PubMed, and Cochrane Library. The search period included January 1, 2010, to July 1, 2025. The first and second authors employed an independent double-blind method to search the literature.

#### 2.1.2 Search strategy

To explore the effects of traditional Chinese exercises on hypertension, blood lipids, and endothelial function in middle-aged and older adults populations, comprehensive searches were conducted using a combination of subject headings and free-text keywords. The Chinese search terms comprised key concepts such as “hypertension,” “blood lipids,” “endothelial function,” “middle-aged and older adults,” “traditional Chinese exercises,” “Tai Chi,” “Baduanjin,” “Wuqinxi,” “Yijinjing,” “randomized controlled trial,” and “randomization”. The English search terms comprised key concepts such as “hypertension,” “blood lipid,” “endothelial function,” “Tai Chi,” “Baduanjin,” “Wuqinxi,” “Yijinjing,” “traditional Chinese exercises,” and “randomized controlled trial.” For example, using Web of Science database, our search strategy was as follows:

#1 ([TI = (hypertension)] OR TI = (Blood lipid)) OR TI = (Endothelial function)
#2 ((((TI = (traditional Chinese exercises)) OR TI = (Tai Chi)) OR TI = (Baduanjin)) OR TI = (Wuqinxi)) OR TI = (Yijinjing)
#3 ((AB = (Randomized controlled trial)) OR AB = (Random)) OR AB = (RCT)
#4 #1 AND #2 AND #3

To streamline the screening process, all records retrieved from the database searches were imported into Zotero for duplicate removal, initial screening, and organization. Two researchers independently screened the literature and cross-verified extracted data. Disagreements were resolved through consultation with a third reviewer.

### 2.2 Inclusion and exclusion criteria

Literature screening was conducted by two independent researchers following the PICOS framework (Population, Intervention, Comparison, Outcomes, Study design). Disagreements between reviewers were resolved through discussion or consultation with a third reviewer.

#### 2.2.1 Population

The research subjects were middle-aged and older adults patients with primary grade 1 and grade 2 stage 1 hypertension.

#### 2.2.2 Intervention

Traditional Chinese exercises, including Tai Chi, Baduanjin, Wuqinxi, and Yijinjing.

#### 2.2.3 Comparison

Control groups that received conventional treatment, no specific intervention, or alternative interventions.

#### 2.2.4 Outcomes

The primary outcomes consisted of blood pressure measurements, specifically systolic blood pressure (SBP) and diastolic blood pressure (DBP), lipid profile parameters, including TC, triglycerides (TG), high-density lipoprotein cholesterol (HDL-C), and low-density lipoprotein cholesterol (LDL-C), and endothelial function indicators, such as endothelin (ET) and nitric oxide (NO).

#### 2.2.5 Study design

Randomized controlled trials that employ parallel or crossover designs.

We included studies with a randomized controlled trial design; a study population that included middle-aged and older adults hypertensive patients; traditional Chinese exercises (Tai Chi, Baduanjin, Wuqinxi, or Yijinjing) as the focus of the experimental interventions; with an intervention that must be completed within at least 6 weeks; and comprehensive data regarding endothelial function indicators, cholesterol parameters, and blood pressure is available. We excluded studies that emphasized non-experimental approaches, such as reviews, observational studies, case reports, and conference abstract; *in vitro* or animal studies; studies that included redundant data or duplicate publications; The research subjects do not include secondary hypertension; they have not experienced major cardiovascular events (such as myocardial infarction, stroke) within the past 6 months; they do not have diseases that affect their exercise capacity (such as severe arthritis, heart failure at NYHA grade III-IV); and they are not participating in other clinical trials simultaneously.

### 2.3 Literature screening

Zotero reference management software (version 7.0.24; Corporation for Digital Scholarship, Fairfax, VA, USA) was utilized to conduct a systematic literature review, with the aim of eliminating duplicates. A two-stage hierarchical screening protocol was implemented by two independent reviewers. Two reviewers separately evaluated the titles and abstracts based on the eligibility criteria, thereafter conducting a full-text review. They documented their decisions separately and had an organized discussion to resolve any disagreements. If there was no agreement, a third senior researcher was consulted to make the final decision. Quality assurance included regular calibration exercises and systematic documentation of exclusion reasons. The initial database search yielded 433 potentially relevant articles, which underwent systematic screening and resulted in 19 studies meeting all inclusion criteria for the final meta-analysis.

The measurement methods for the included indicators:

Blood pressure measurement: Before and after the intervention, the blood pressure of the patients in both groups was measured using a standard mercury column sphygmomanometer. Before measuring the blood pressure, the patients were required to sit quietly for at least 5 min, keep their emotions stable, and refrain from smoking or drinking tea. The patients sat on a chair, fully exposing their upper arms, and placed the upper arms at the same level as the heart.

Blood lipid indicators were tested using the enzyme-linked immunosorbent assay (ELISA).

Endothelin determination: Using radioimmunoassay. Before and after the intervention, 2 mL of fasting venous blood was taken from each group of patients and mixed with 30 μL of 100 g/L disodium ethylenediaminetetraacetate and 400 IU of aprotinin in a test tube. The mixture was centrifuged and the plasma was separated. The samples were aliquoted and stored at −40 °C for testing. The radioimmunoassay operation was strictly carried out according to the requirements of the kit. The intra-batch coefficient of variation of the kit was 3.6%, and the inter-batch coefficient of variation was 6.5%. The radioimmunoassay kit for endothelin and the monitor instrument were supplied by a specialized unit.

NO determination: Before and after the intervention, 2 mL of fasting venous blood was taken from each group of patients and placed in dry tubes, centrifuged, and the serum was stored at −40 °C. The determination was carried out according to the requirements of the kit. The standard curve was prepared using nitrite ion standard solution. The intra-batch coefficient of variation was 2.7%, and the inter-batch coefficient of variation was 5.3%. NO reagents and monitoring instruments are supplied by specialized units.Assessment of risk of literature bias

Methodological quality assessment of included studies was performed using the Cochrane Risk of Bias assessment tool implemented in RevMan 5.3 software. Study quality was systematically evaluated across six key domains: random sequence generation (selection bias), allocation concealment (selection bias), blinding of participants and personnel (performance bias), blinding of outcome assessment (detection bias), incomplete outcome data (attrition bias), and selective reporting (reporting bias). Two independent investigators conducted the bias assessment for all included studies. Based on the information provided in the original publications, each study was assigned a low, high, or unclear risk of bias for each domain. Any disputes between the two reviewers were handled through conversation; if consensus could not be reached, a third reviewer was consulted to determine the final decision. This methodical approach ensured that the quality assessment procedure was reliable and objective.

### 2.4 Data extraction

Two reviewers used a standardized extraction form to collect data from eligible studies, including study identification information (first author, publication year), participant characteristics (sample size, mean age, disease duration), intervention details (exercise type, frequency, duration, intervention period), and outcome measurements (blood pressure, lipid profile, and endothelial function parameters with means and standard deviations). To guarantee precision, all data were systematically organized in Excel spreadsheets and verified by reviewers. Discrepancies were resolved through discussion, and a third reviewer conducted quality checks to verify data integrity and consistency across studies.

### 2.5 Statistical analysis

This study employed RevMan 5.3 software for quality assessment of the included studies. Stata 15.1 software was used to conduct network meta-analysis for multiple outcome indicators including blood pressure (SBP, DBP), lipid profile parameters (TC, TG, HDL-C, LDL-C), and endothelial function markers (NO, ET). Standardized mean difference (MD) with 95% confidence interval (CI) was used as the effect analysis statistic for all comparisons. Statistical significance was set at *p* < 0.05. Network evidence diagrams were constructed to visualize direct and indirect comparisons among the four traditional Chinese exercise interventions (Tai Chi, Baduanjin, Wuqinxi, Yijinjing) and conventional treatment. Node size in the network diagrams represented sample size, while edge thickness indicated the number of studies with direct comparisons. Since no closed loops were formed among the outcome indicators in the network, consistency testing was not performed. The Surface Under the Cumulative Ranking curve (SUCRA) probability rankings were computed for numerous traditional Chinese exercise regimens across multiple outcome variables, with higher SUCRA values indicating more efficacious interventions. SUCRA ranking curves were created to visually depict these probability rankings. The assessment of publication bias was conducted using funnel plots for all outcome measures. Asymmetry in the scatter of data points was considered indicative of potential publication bias or small-study effects.

## 3 Results

### 3.1 Literature screening process and results

**Figure 1:**
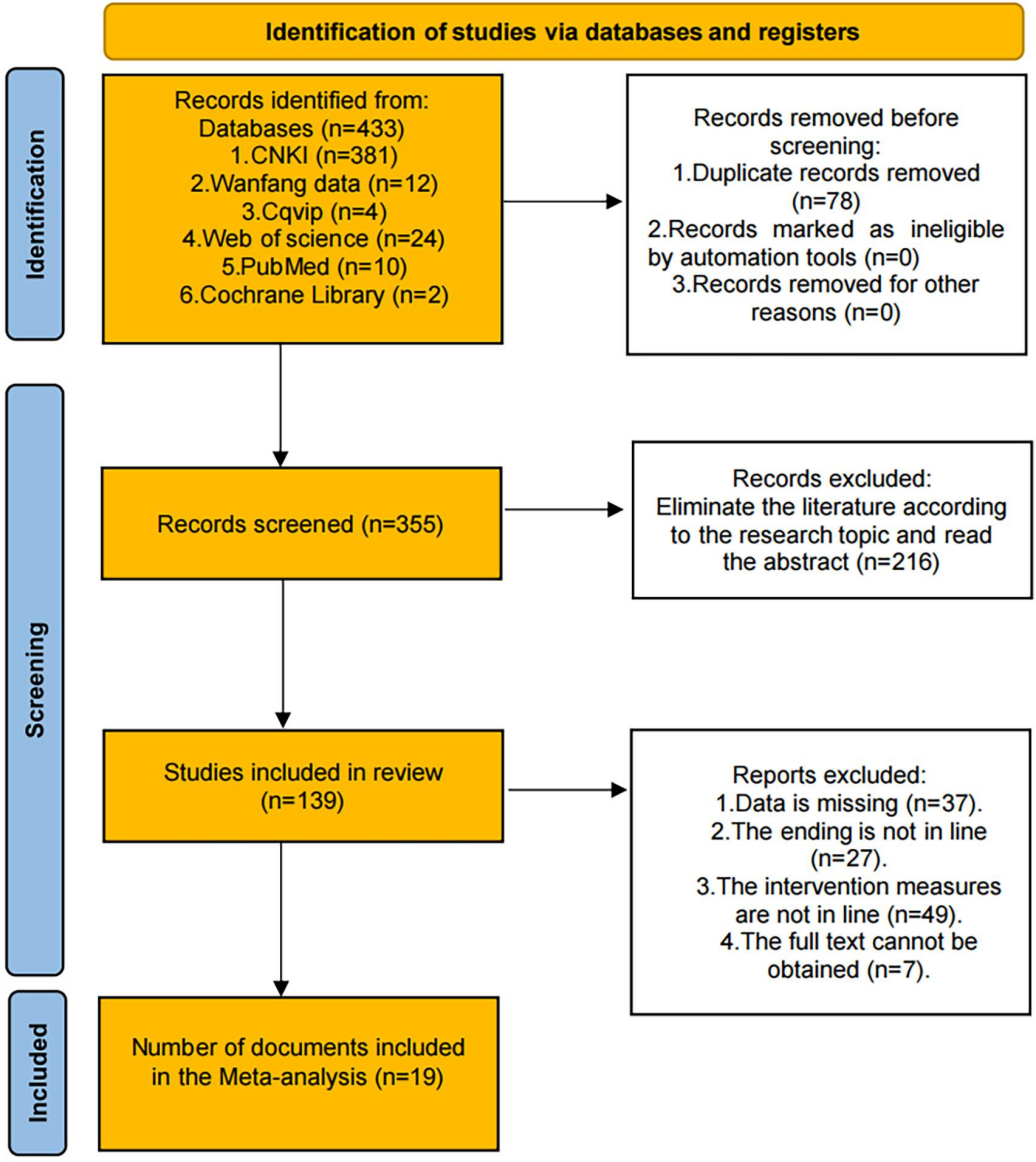
PRISMA flow diagram of literature screening process.

The comprehensive literature search across six databases initially identified 433 articles (CNKI: 381 articles, Wanfang data: 12 articles, Cqvip: 4 articles, Web of Science: 24 articles, PubMed: 10 articles, and Cochrane Library: 2 articles). After removing 78 duplicate records, 355 articles remained for the first screening. During the screening phase, 216 papers were removed due to a review of their titles and abstracts, as they did not conform to the research topic and inclusion criteria. The remaining 139 studies underwent full-text assessment for eligibility. Subsequently, 120 studies were excluded for the following reasons: insufficient or missing data (*n* = 37), outcomes not meeting the research requirements (*n* = 27), intervention methods not conforming to study criteria (*n* = 49), and inability to obtain full-text access (*n* = 7). Ultimately, 19 studies met all inclusion criteria and were incorporated into the meta-analysis. The detailed literature screening process and results are illustrated in Figure 1.

### 3.2 Basic characteristics of included studies

This network meta-analysis includes 1,501 participants from 19 randomized controlled trials, with 772 assigned to the experimental group and 729 to the control group. Not all studies reported all outcome indicators: 18 studies reported blood pressure outcomes (*n* = 1,441), nine measured lipid profiles, and four measured endothelial function markers. The experimental interventions encompassed traditional Chinese exercises, including Tai Chi, Baduanjin, Wuqinxi, and Yijinjing. The training frequencies varied from three times per week to daily sessions, and the intervention duration ranged from 6 to 48 weeks. Meanwhile, control groups received conventional medical treatment or standard care. The baseline characteristics and methodological details of the included studies are presented in Table 1.

**Table 1:** Baseline characteristics and methodological details of included studies.

Included study literature (First author)	Experimental group				Control group				Time and frequency of intervention	Intervention duration	Outcome index
	N	Age/years	Disease duration	Intervention	N	Age/ years	Disease duration	Intervention			
Feng Lijuan 2018 (47)	37	67.51 ± 4.09	-	1 + F	36	66.33 ± 4.74	-	*N* + F	1 h/session, 3 sessions/week	12 weeks	SBP, DBP, TC, TG, HDL-C, LDL-C
Hu Lixun 2015 (48)	55	64.21 ± 6.12	4.92 ± 2.65	1 + F	55	64.10 ± 7.03	5.12 ± 2.45	N + F	45 min/session, 5 sessions/week	12 weeks	SBP, DBP, TC, TG, HDL-C, LDL-C
Zheng Yongcai 2015 (49)	49	54.71 + 5.43	8 ± 2.44	1 + F	49	55.77 + 6.24	7 + 1.73	F	40–60 min/session, 4–8 sessions/week	12 weeks	SBP, DBP, TC, TG, HDL-C, LDL-C
Jin Haolei 2016 (50)	27	-	-	1	27	-	-	F	40 min/day	6 weeks	SBP, DBP, ET, NO
Liu Wenqi 2016 (51)	30	56.33 ± 7.16	14.20 ± 6.88	1 + F	30	56.80 ± 6.78	13.23 ± 7.56	N + F	40 min/day, 1 session/day, 5 sessions/week	12 weeks	ET, NO
Mao Hongni 2016 (52)	51	62.2	-	1 + F	11	63.3	-	N + F	1 h/session, 6 sessions/week	8 weeks	SBP, DBP, ET, NO
Pan Huashan 2010 (53)	24	62.1 ± 5.8	1.5 ± 1.2	2 + F	24	61.4 ± 7.1	1.7 ± 0.8	F	45 min/session, 10 sessions/week	24 weeks	SBP, DBP, TC, TG, HDL-C
Cao Chen 2021 (54)	112	60.89 ± 9.70	-	2 + N	114	61.33 ± 8.23	-	N	5 days/week, 1 session/day, 30-40 min/session	48 weeks	SBP, DBP
Wei Wei 2017 (55)	20	-	-	2	20	-	-	N + F	45 min/session, 12 sessions/week	24 weeks	SBP, DBP, TC, TG, HDL-C, LDL-C
Liang Yunhua 2014 (56)	30	54.8 ± 7.6	4.3 ± 3.0	2 + N	30	55.7 ± 8.8	4.7 ± 3.2	N	10 sessions/week, 20 min/session	24 weeks	SBP, DBP, TC, TG, HDL-C, LDL-C
Liao Seqing 2013 (57)	70	60.5 ± 11.8	4.8 ± 2.1	2	70	62.7 ± 9.5	3.9 ± 3.1	N + F	Twice daily (morning and evening), 1 session each time, 30 min/session	24 weeks	SBP, DBP, TC, TG
Chen Qingyue 2013 (58)	30	69.23 ± 3.72	9.13 ± 3.69	2 + F	30	70.06 ± 3.51	8.30 ± 4.36	N + F	1 session/day, 30 min/session, 5 sessions/week	12 weeks	SBP, DBP, ET, NO
Xin Sicong 2016 (59)	16	62.43 ± 6.71	-	3	17	62.11 ± 6.81	-	N	60 min/session, 3 sessions/week	12 weeks	SBP, DBP
Niu Yaru 2023 (60)	31	44.19 ± 7.86	-	3 + N	30	46.67 ± 7.53	-	N	3 sessions each time (morning and evening), 5 days/week	12 weeks	SBP, DBP
Zhou Huihui 2022 (61)	43	35-70 years	5 months-11 years	3 + F	43	31-70 years	6 months-12 years	N + F	40 min/session, 5 sessions/week	12 weeks	SBP, DBP
Yuan Zhaoxia 2011 (62)	57	-	-	3	53	-	-	N	5 sessions/week, 60 min/session	12 weeks	SBP, DBP, TC, TG, HDL-C, LDL-C
Hong Hao 2017 (13)	25	-	-	4	25	-	-	N	7 sessions/week, 90 min/session	12 weeks	SBP, DBP
Yao Bingke 2017 (63)	30	45.89 ± 6.36	-	4 + N	30	45.23 ± 5.76	-	N	7 sessions/week, 90 min/session	12 weeks	SBP, DBP
Su Yufeng 2012 (64)	35	-	-		35	-	-	N	-	12 weeks	SBP, DBP, TC, TG, HDL-C, LDL-C
“-” = not reported; N, Conventional treatment(Nursing + Walking);F,Drug treatment;; Interventions: 1, Tai Chi; 2, Baduanjin; 3, Wuqinxi; 4, Yijinjing.											

Included study literature (First author)	Experimental group (Pre-experiment assessment)		Control group (Pre-experiment assessment)		Experimental group (Post-experiment assessment)		Control group (Post-experiment assessment)	
	SBP(mmHg)	DBP(mmHg)	SBP(mmHg)	DBP(mmHg)	SBP(mmHg)	DBP(mmHg)	SBP(mmHg)	DBP(mmHg)
Feng Lijuan 2018 (47)	144.17 ± 8.96	81.39 ± 8.67	145.22 ± 11.31	81.89 ± 8.88	133.42 ± 7.69	78.86 ± 7.32	143.46 ± 11.50	81.05 ± 7.33
Hu Lixun 2015 (48)	152 ± 22	92 ± 14	154 ± 21	90 ± 12	130 ± 20	76 ± 12	144 ± 20	82 ± 16
Zheng Yongcai 2015 (49)	164.51 ± 10.92	101.11 ± 4.22	161.41 ± 10.61	99.42 ± 8.32	124.48 ± 8.71	75.32 ± 3.84	136.43 ± 9.65	84.73 ± 7.84
Jin Haolei 2016 (50)	152.44 ± 16.21	92.92 ± 14.35	150.24 ± 11.36	140.97 ± 10.09	137.82 ± 11.65	86.99 ± 11.14	97.19 ± 9.35	87.64 ± 9.76
Liu Wenqi 2016 (51)	/	/	/	/	/	/	/	/
Mao Hongni 2016 (52)	162.74 ± 26.36	93.66 ± 13.72	161.72 ± 64.96	94.09 ± 38.71	140.49 ± 23.55	82.74 ± 11.97	163.89 ± 67.63	94.35 ± 38.90
Pan Huashan 2010 (53)	145.2 ± 3.7	94.5 ± 4.1	144.6 ± 3.9	83.7 ± 5.4	125.4 ± 6.2	95.3 ± 3.4	134.8 ± 4.5	88.7 ± 5.1
Cao Chen 2021 (54)	152.14 ± 7.31	96.24 ± 5.44	151.89 ± 6.51	95.66 ± 6.03	145.67 ± 7.77	93.71 ± 5.77	149.13 ± 7.39	95.63 ± 5.32
Wei Wei 2017 (55)	145.64 ± 5.34	93.69 ± 5.46	146.14 ± 6.54	94.53 ± 4.68	135.34 ± 9.54	86.26 ± 4.87	142.49 ± 5.87	92.21 ± 3.79
Liang Yunhua 2014 (56)	158.6 ± 12.3	101.7 ± 8.5	160.1 ± 11.7	100.9 ± 9.1	136.4 ± 10.4	85.1 ± 7.5	145.7 ± 12.3	89.5 ± 7.3
Liao Seqing 2013 (57)	146.5 ± 9.5	96.7 ± 7.5	148.2 ± 10.3	95.8 ± 7.6	127.8 ± 8.1	83.1 ± 6.4	135.9 ± 7.2	8.6 ± 6.8
Chen Qingyue 2013 (58)	141.93 ± 6.10	80.53 ± 7.75	139.80 ± 6.75	80.57 ± 8.11	128.63 ± 5.04	74.70 ± 5.18	131.68 ± 6.16	77.50 ± 6.26
Xin Sicong 2016 (59)	149.81 ± 11.30	83.68 ± 7.47	148.29 ± 12.40	84.82 ± 4.18	139.43 ± 11.15	78.75 ± 7.14	148.23 ± 12.58	84.76 ± 4.05
Niu Yaru 2023 (60)	147.20 ± 3.72	94.71 ± 2.20	147.50 ± 4.18	94.53 ± 2.29	128.81 ± 2.75	84.13 ± 2.23	135.40 ± 2.42	88.20 ± 2.20
Zhou Huihui 2022 (61)	159.14 ± 3.20	84.12 ± 3.20	140.23 ± 3.19	83.21 ± 3.21	125.21 ± 3.30	69.12 ± 3.20	133.21 ± 3.21	83.21 ± 3.21
Yuan Zhaoxia 2011 (62)	122.70 ± 10.05	75.84 ± 6.54	122.60 ± 11.96	75.53 ± 8.48	116.93 ± 8.32	72.79 ± 8.56	121.72 ± 12.27	76.64 ± 8.01
Hong Hao 2017 (13)	134.08 ± 8.87	88.32 ± 5.99	131.88 ± 6.61	89.56 ± 4.88	119.96 ± 6.33	80.40 ± 4.11	127.64 ± 6.22	87.08 ± 3.75
Yao Bingke 2017 (63)	140.23 ± 7.86	89.54 ± 6.30	139.15 ± 6.75	89.69 ± 3.89	123.69 ± 6.16	81.15 ± 4.39	134.96 ± 7.51	88.54 ± 3.54
Su Yufeng 2012 (64)	129.23 ± 7.98	86.74 ± 7. 32	132.54 ± 10.21	86.32 ± 5.37	122.13 ± 9.67	82.31 ± 4.32	131.56 ± 9.45	84.21 ± 6.33

The absence of blood pressure data is due to the fact that the original literature directly reported the standard deviations of dynamic blood pressure for both groups before and after treatment, but did not provide the basic data of systolic and diastolic blood pressure.

### 3.3 Bias analysis

**Figure 2:**
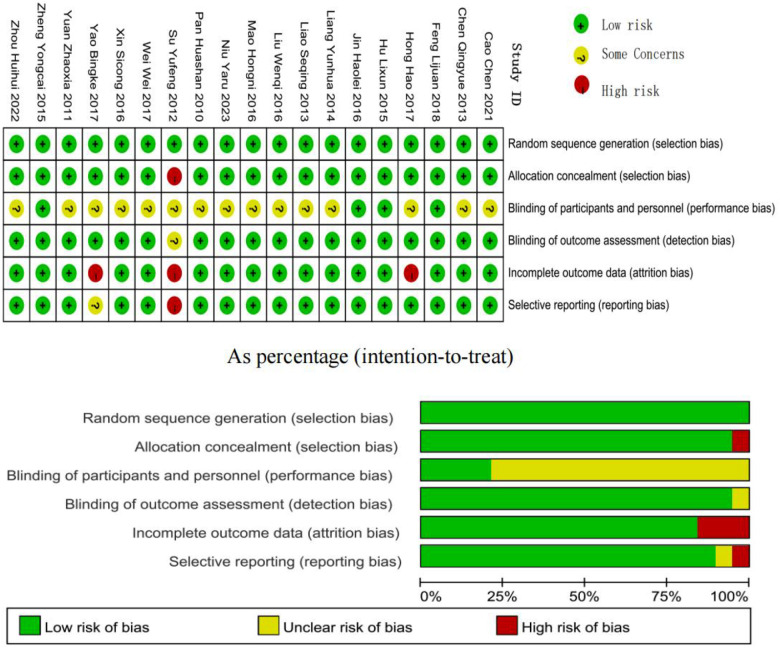
Risk of bias assessment of included studies.

The quality assessment of the included studies was conducted across the six domains (Figure 2). Among the 19 included studies, four demonstrated high methodological quality, 12 presented some risk, and three were classified as high risk. Of the 114 total assessment items, 92 were rated as low risk, 17 as unclear risk, and five as high risk. No studies showed randomization concerns, while 15 studies presented risks related to deviations from intended interventions, three studies had certain outcome data that could not be extracted (although other outcome data were accessible), one study exhibited risks in outcome measurement, and two studies demonstrated potential publication bias.

### 3.4 Network meta-analysis results

#### 3.4.1 Network evidence

**Figure 3:**
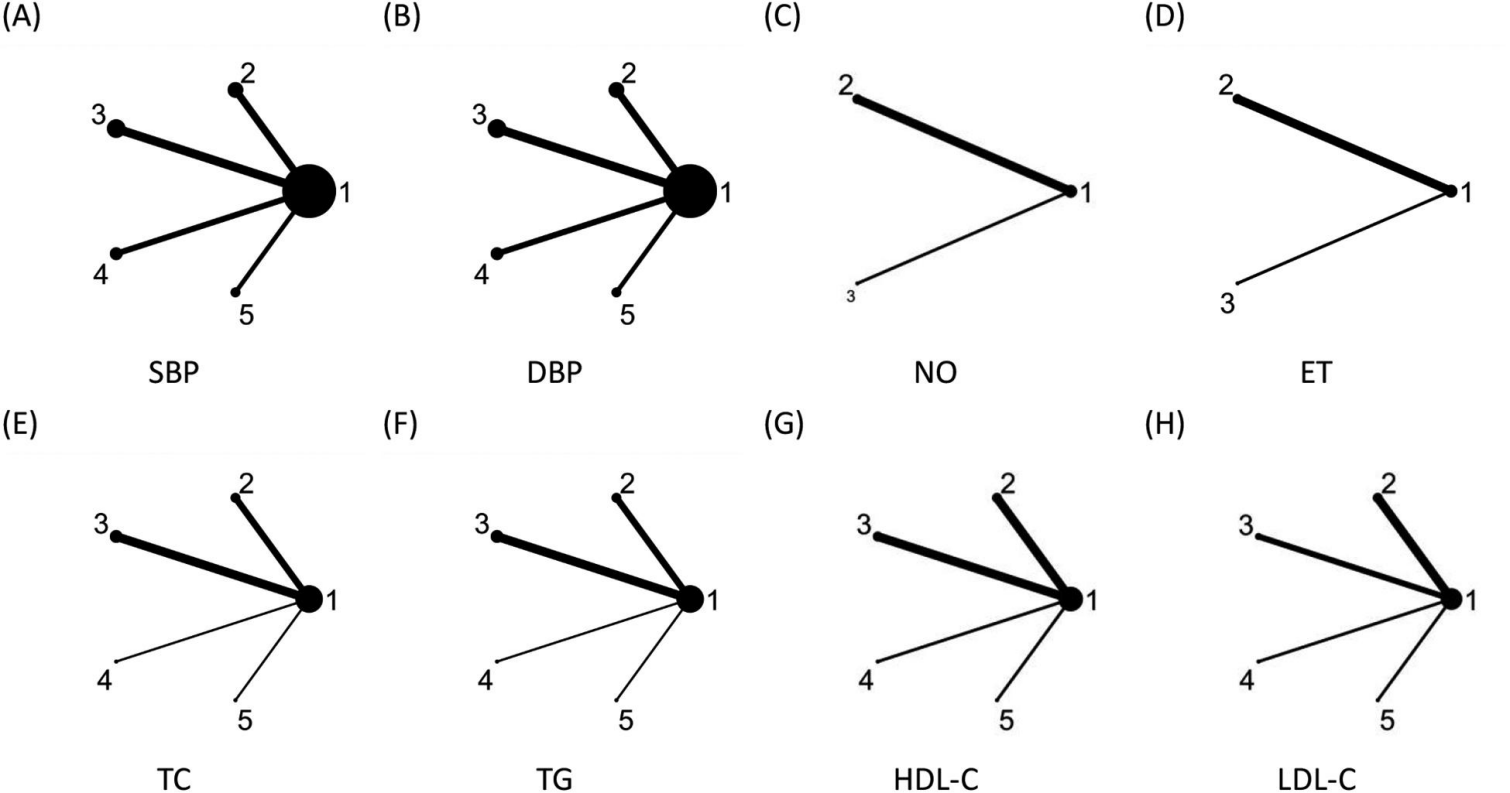
Network evidence diagrams for different outcome indicators. 1: Control group (conventional treatment); 2: Tai Chi intervention; 3: Baduanjin intervention; 4: Wuqinxi intervention; 5: Yijinjing intervention.

The studies included in this research involved four types of traditional Chinese exercises: Tai Chi, Baduanjin, Wuqinxi, and Yijinjing. For blood pressure indicators, 18 studies reported SBP and 18 studies reported DBP, each forming four direct comparisons. For lipid profile indicators, nine studies reported TC, nine studies reported TG, eight studies reported HDL-C, and seven studies reported LDL-C, each forming four direct comparisons. For endothelial function indicators, four studies reported nitric oxide (NO) and four studies reported endothelin (ET), each forming two direct comparisons (Figure 3). Node size represents sample size, while edge thickness indicates the number of studies with direct comparisons. Since no closed loops were formed among the outcome indicators, consistency testing was not performed.

#### 3.4.2 Blood pressure indicators

Among the 18 studies that included SBP indicators, a total of 1,441 participants were involved. The comparative results showed that four traditional Chinese exercises: Tai Chi (MD = −6.06, 95% CI [−9.92, −2.20], Yijinjing (MD = −5.36, 95% CI [−9.84, −0.89]), Wuqinxi [MD = −3.85, 95% CI (−7.77, 0.07)], and Baduanjin [MD = −3.71, 95% CI (−6.94, −0.49)], were superior to conventional treatment in improving SBP in middle-aged and older adults hypertensive patients (Table 2). However, no significant difference was observed between Wuqinxi [MD = −3.85, 95% CI (−7.77, 0.07)] and conventional treatment.

**Table 2:** Network meta-analysis results: standardized mean differences for SBP and DBP.

Indicator	Network meta-analysis results				
SBP	Tai Chi				
	−0.70 (−6.60, 5.21)	Yijinjing			
	−2.21 (−7.71, 3.29)	−1.51 (−7.46, 4.44)	Wuqinxi		
	−2.35 (−7.37, 2.68)	−1.65 (−7.16, 3.86)	−0.14 (−5.21, 4.93)	Baduanjin	
	−6.06 (−9.92, −2.20)	−5.36 (−9.84, −0.89)	−3.85 (−7.77, 0.07)	−3.71 (−6.94, −0.49)	Conventional treatment
DBP	Tai Chi				
	−0.48 (−4.96, 4.00)	Yijinjing			
	−3.04 (−7.08, 1.01)	−2.56 (−6.57, 1.45)	Wuqinxi		
	−3.62 (−7.46, 0.22)	−3.14 (−6.93, 0.65)	−0.58 (−3.86, 2.70)	Baduanjin	
	−9.94 (−13.13, −6.75)	−9.46 (−12.61, −6.32)	−6.91 (−9.40, −4.42)	−6.32 (−8.44, −4.20)	Conventional treatment

Among the 18 studies that included DBP indicators, a total of 1,441 participants were involved. Regarding the effects of the four traditional Chinese exercise interventions on improving DBP in middle-aged and older adults hypertensive patients, Tai Chi [MD = −9.94, 95% CI (−13.13, −6.75)], Yijinjing [MD = −9.46, 95% CI (−12.61, −6.32)], Wuqinxi [MD = −6.91, 95% CI (−9.40, −4.42)], and Baduanjin [MD = −6.32, 95% CI (−8.44, −4.20)] were all superior to conventional treatment, with statistically significant differences (Table 2).

#### 3.4.3 Lipid profile indicators

Nine studies included TC investigations, involving 935 participants. The comparative results showed that Baduanjin [MD = −0.89, 95% CI (−1.27, −0.52)] and Tai Chi [MD = −0.51, 95% CI (−0.92, −0.10)] were superior to conventional treatment with statistically significant differences (Table 3), while Wuqinxi [MD = −0.18, 95% CI (−0.85, 0.49)] showed no significant difference from conventional treatment.

**Table 3:** Network meta-analysis results: standardized mean differences for TC, TG, HDL-C, and LDL-C.

Indicator	Network meta-analysis results				
TC	Baduanjin				
	−0.39 (−0.94, 0.17)	Tai Chi			
	−0.71 (−1.48, 0.06)	−0.33 (−1.11, 0.46)	Wuqinxi		
	−0.98 (−1.83, −0.13)	−0.60 (−1.46, 0.27)	−0.27 (−1.29, 0.75)	Yijinjing	
	−0.89 (−1.27, −0.52)	−0.51 (−0.92, −0.10)	−0.18 (−0.85, 0.49)	0.09 (−0.67, 0.85)	Conventional treatment
TG	Tai Chi				
	−0.36 (−1.00, 0.29)	Baduanjin			
	−0.63 (−1.56, 0.31)	−0.27 (−1.17, 0.63)	Yijinjing		
	−0.74 (−1.65, 0.18)	−0.38 (−1.25, 0.49)	−0.11 (−1.21, 0.99)	Wuqinxi	
	−0.83 (−1.33, −0.33)	−0.47 (−0.88, −0.06)	−0.20 (−0.99, 0.59)	−0.09 (−0.86, 0.68)	Conventional treatment
HDL-C	Tai Chi				
	−1.58 (−4.95, 1.78)	Baduanjin			
	−1.73 (−6.49, 3.02)	−0.15 (−4.91, 4.61)	Wuqinxi		
	−1.89 (−6.65, 2.87)	−0.31 (−5.07, 4.46)	−0.16 (−5.99, 5.67)	Yijinjing	
	−1.59 (−3.97, 0.79)	−0.01 (−2.39, 2.38)	0.14 (−3.98, 4.26)	0.30 (−3.82, 4.42)	Conventional treatment
LDL-C	Tai Chi				
	0.02 (−1.35, 1.39)	Baduanjin			
	−0.14 (−1.88, 1.60)	−0.17 (−2.01, 1.68)	Yijinjing		
	−0.23 (−1.96, 1.50)	−0.26 (−2.08, 1.57)	−0.09 (−2.21, 2.03)	Wuqinxi	
	−0.49 (−1.36, 0.38)	−0.52 (−1.57, 0.54)	−0.35 (−1.86, 1.16)	−0.26 (−1.75, 1.23)	Conventional treatment

Nine studies investigated TG, involving 935 participants. The comparative results showed that regarding the effects of four traditional Chinese exercise interventions on improving triglycerides (TG) in middle-aged and older adults hypertensive patients, Tai Chi [MD = −0.83, 95% CI (−1.33, −0.33)], Baduanjin [MD = −0.47, 95% CI (−0.88, −0.06)], Yijinjing [MD = −0.20, 95% CI (−0.99, 0.59)], and Wuqinxi [MD = −0.09, 95% CI (−0.86, 0.68)] were all superior to conventional treatment (Table 3). However, no significant differences were observed between Yijinjing, Wuqinxi, and conventional treatment.

Eight studies were included in the HDL-C research, involving 795 participants. The network meta-analysis results showed that Tai Chi [MD = −1.59, 95% CI (−3.97, 0.79)], Baduanjin [MD = −0.01, 95% CI (−2.39, 2.38)], Wuqinxi [MD = 0.14, 95% CI (−3.98, 4.26)], and Yijinjing [MD = 0.30, 95% CI (−3.82, 4.42)] showed intervention effects compared to conventional treatment. However, these differences did not reach statistical significance (Table 3).

Seven studies, involving 747 participants, were incorporated into the LDL-C research. The network meta-analysis results showed that Tai Chi [MD = −0.49, 95% CI (−1.36, 0.38)], Baduanjin [MD = −0.52, 95% CI (−1.57, 0.54)], Wuqinxi [MD = −0.35, 95% CI (−1.86, 1.16)], and Yijinjing [MD = −0.26, 95% CI (−1.75, 1.23)] demonstrated superior intervention effects compared to conventional treatment. However, no significant differences were observed between the four traditional Chinese exercises and conventional treatment (Table 3).

#### 3.4.4 Endothelial function indicators

The research on NO encompassed four studies with a combined total of 236 participants. The results of the network meta-analysis indicated that both Tai Chi [MD = 9.52, 95% CI (−-3.03, 22.06)] and Baduanjin [MD = 2.82, 95% CI (−18.30, 23.94)] interventions showed a trend toward increasing serum NO levels compared to conventional treatment, although these differences did not reach statistical significance (Table 4). Four studies were included in the serum ET research, involving 236 participants. The network meta-analysis results showed that Tai Chi [MD = −10.72, 95% CI (−21.52, 0.07)] and Baduanjin [MD = −6.64, 95% CI (−19.92, 6.64)] interventions were superior to conventional treatment in improving serum ET in middle-aged and older adults hypertensive patients (Table 4).

**Table 4:** Network meta-analysis results: standardized mean differences for NO and ET.

Indicator	Network meta-analysis results		
NO	Tai Chi		
	6.70 (−17.87, 31.26)	Baduanjin	
	9.52 (−3.03, 22.06)	2.82 (−18.30, 23.94)	Conventional treatment
ET	Tai Chi		
	−4.08 (−21.20, 13.03)	Baduanjin	
	−10.72 (−21.52, 0.07)	−6.64 (−19.92, 6.64)	Conventional treatment

### 3.5 SUCRA probability rankings for network meta-analysis

**Figure 4:**
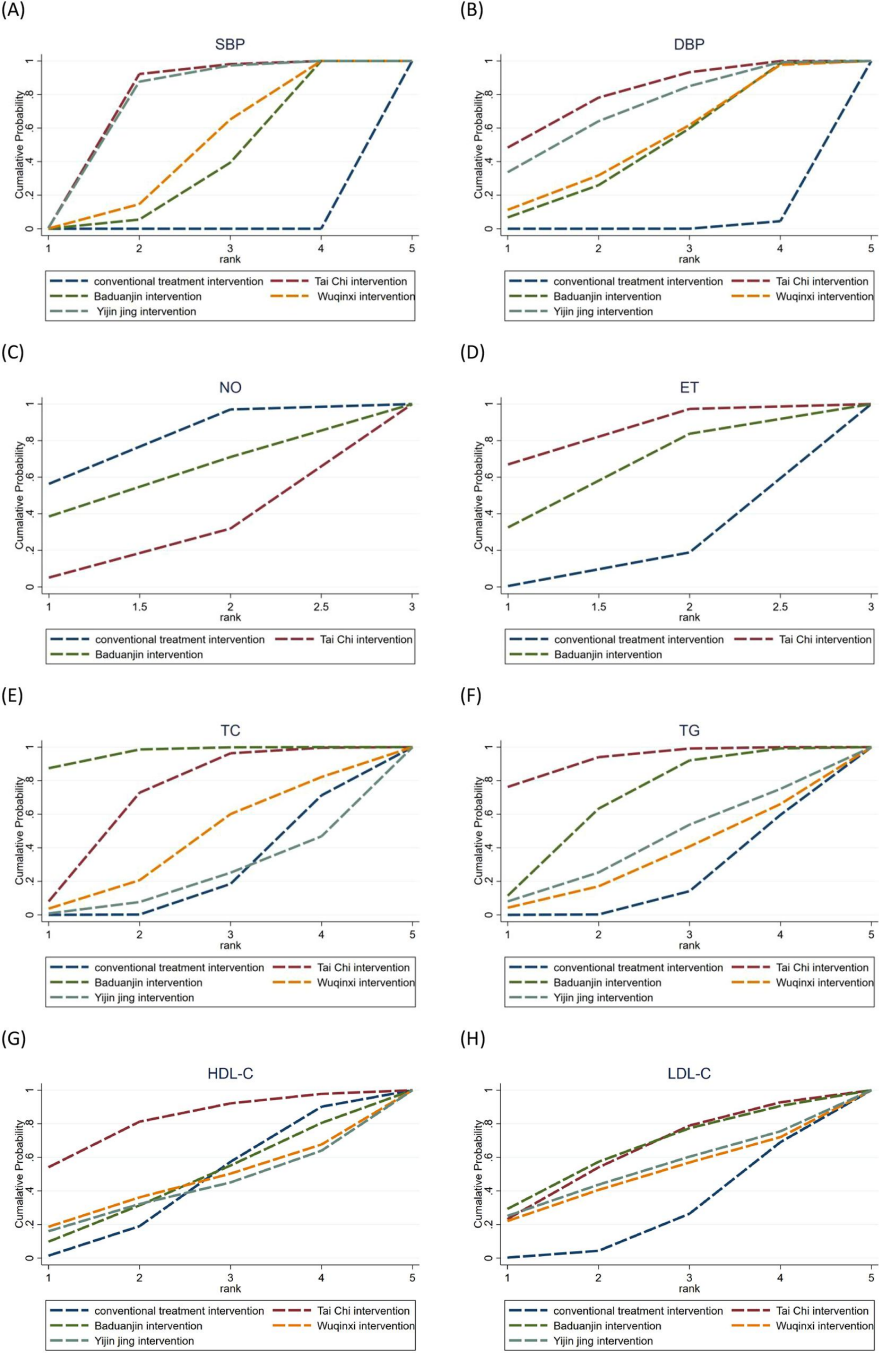
SUCRA ranking curve for different traditional Chinese exercise intervention across multiple outcome indicators.

The probability rankings for various traditional Chinese exercise interventions aimed at enhancing blood pressure, lipid profile, and endothelial function in middle-aged and older adults hypertensive patients were predominantly determined by SUCRA values, with elevated SUCRA rankings signifying more effective interventions. The SUCRA ranking findings (Table 5) and ranking curve diagrams (Figure 4) indicate that Tai Chi exhibited superior efficacy in enhancing systolic and DBP in middle-aged and older adults hypertensive patients, with SUCRA values of 86.7% and 79.3%, respectively. Baduanjin (54.8%) and Tai Chi (82.2%) demonstrated superior benefits on blood NO and ET markers of endothelial function in middle-aged and older adults hypertensive patients. For TC, TG, HDL-C, and LDL-C, the optimal interventions were Baduanjin (96.5%), Tai Chi (92.4%), Tai Chi (81.3%), and Baduanjin (63.6%), respectively.

**Table 5:** SUCRA probability rankings for different traditional Chinese exercise interventions across outcome indicators.

Exercise Intervention	SBP	DBP	NO	ET	TC	TG	HDL-C	LDL-C
Tai Chi	86.7	79.3	18.5	82.2	69.2	92.4	81.3	62.2
Baduanjin	36.3	48.4	54.8	58.1	96.5	66.5	44.2	63.6
Wuqinxi	45.5	51.1	-	-	41.7	32.1	43.2	47.9
Yijinjing	81.4	70.1	-	-	20.1	40.6	39.3	51.2

“-” means not reported.

### 3.6 Publication bias assessment

**Figure 5:**
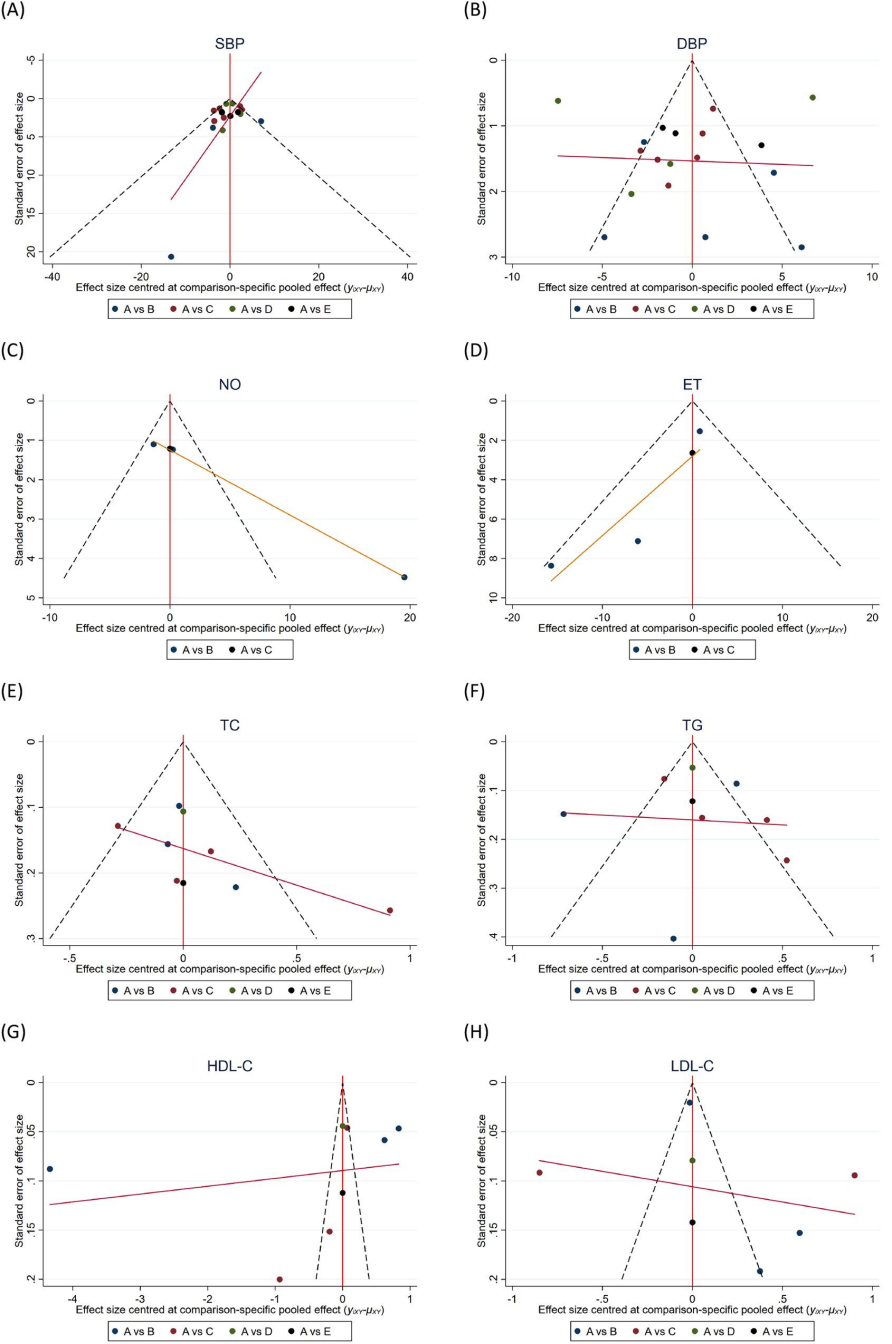
Funnel plots for publication bias assessment across all outcome indicators.

Using SBP, DBP, NO, ET, TC, TG, HDL-C, and LDL-C from the included studies as outcome indicators, the constructed funnel plots showed asymmetric distribution of scattered points within the funnel plot range, all with slopes indicating the possibility of publication bias or small sample effects in the studies, suggesting potential publication bias and small sample effects in the literature (Figure 5).

## 4 Discussion

We systematically evaluated the effects of four traditional Chinese exercises (Tai Chi, Baduanjin, Wuqinxi, and Yijinjing) on cardiovascular metabolic indicators (SBP, DBP; TC, TG, HDL-C, LDL-C; ET, NO) in middle-aged and older adults hypertensive patients through network meta-analysis of 19 included randomized controlled trials. Compared with conventional treatment, different traditional Chinese exercises improved cardiovascular metabolic function in middle-aged and older adults hypertensive patients to varying degrees, with distinct differences in improvement effects among the exercises.

The SUCRA probability ranking results revealed that Tai Chi exhibited the most significant enhancement in cardiovascular metabolic indicators such as SBP, DBP, ET, TG, and HDL-C, whereas Baduanjin excelled in enhancing NO, TC, and LDL-C indicators. These results are consistent with prior investigations (11, 19). Traditional Chinese exercise modalities have increasingly attracted international attention in cardiovascular disease prevention and management; multiple studies document efficacy in lowering blood pressure and improving overall cardiovascular function (20–22). Among the exercises, Tai Chi and Baduanjin have proven particularly beneficial for middle-aged and older adults, demonstrating significant reductions in blood pressure alongside improvements in cardiopulmonary endurance, with Tai Chi showing the most robust effects (22, 23).

### 4.1 Physiological mechanisms of different traditional Chinese exercise interventions

The four traditional Chinese exercises improve cardiovascular metabolic function through multidimensional, multi-target synergistic actions. According to current evidence, their mechanisms of action predominantly encompass the following three elements.

#### 4.1.1 Mechanical modulation of vascular function

Exercise-induced hemodynamic changes enhance vascular function by increasing shear stress and promoting endothelial adaptation (24). These mechanisms operate effectively during Tai Chi practice, which consists of sustained, low-to-moderate intensity movements. Practitioners perform coordinated limb motions that pivot around the waist while engaging in deep abdominal breathing. This distinctive movement pattern increases vascular shear stress via two key pathways: first, continuous weight shifts and rhythmic muscle contractions enhance venous return and cardiac output; second, thoracic-abdominal pressure fluctuations facilitate circulatory flow. The consequent increases in blood flow and shear stress stimulate endothelial NO release, which reduces vascular tone and peripheral resistance (24, 25). This cascade improves systemic circulation, benefiting coronary, cerebral, and peripheral micro-vascular perfusion.

#### 4.1.2 Regulation of the neuroendocrine system

As a mind-body integrative exercise form, Tai Chi's practice characteristics of “relaxed naturalness and mental guidance” can effectively regulate autonomic nervous system function. Through meditative mental control and slow, deep breathing, Tai Chi activates parasympathetic nervous system activity while suppressing excessive sympathetic excitation. This re-balancing of the nervous system improves cardiovascular metabolism through two pathways. First, within the blood pressure control pathway, reduced sympathetic nervous system activity directly decreases the secretion of catecholamines (epinephrine and norepinephrine). This reduction subsequently lowers heart rate, weakens myocardial contractility, and dilates peripheral vessels, ultimately decreasing peripheral vascular resistance and reducing blood pressure (26). Second, through the lipid metabolism improvement pathway, optimized neuroendocrine regulation enhances metabolism by increasing lipoprotein activity to accelerate TG-rich lipoprotein breakdown (27) and reducing oxidative stress to decrease lipid per-oxidation damage (28). Regular Tai Chi practice can significantly reduce TG, LDL-C, and atherogenic index while elevating HDL-C levels, with the latter accelerating the clearance of cholesterol deposited in the vascular lumen and walls through the “reverse cholesterol transport” mechanism (29).

#### 4.1.3 Regulation of vascular endothelial function

Vascular endothelial function is one of the primary indicators of cardiovascular metabolism. The vascular endothelium is a mono-layer of flat cells lining the vascular lumen that exerts precise regulatory control over vascular tone and vascular smooth muscle growth through the secretion of vasodilators (such as NO) and vasoconstrictors (such as ET) (30). The dynamic balance between these two categories of vasoactive mediators is essential to maintain vascular homeostasis (31). An imbalance between NO and ET in the body leads to endothelial dysfunction, which is marked by decreased NO bio-availability and increased ET activity (32). Endothelial dysfunction is an early critical event in the pathogenesis of cardiovascular disease, and endothelial cells are essential for maintaining vascular health by regulating vasomotor function, inflammatory responses, and thrombotic processes (33). Enhancing endothelial function is a vital goal for the prevention and management of hypertension and related cardiovascular disorders.

Exercise leads to improved endothelial function by enhancing the phosphorylation of endothelial nitric oxide synthase (eNOS) and increasing NO bio-availability (34). Baduanjin markedly increases serum NO levels in individuals with hypertension (35). The mechanism shows that during exercise, the redistribution of blood flow, especially to skeletal and cardiac muscles, results in heightened vascular shear stress. Shear stress increases NO production through both acute eNOS activation and chronic up-regulation of eNOS expression (36).

Traditional Chinese exercises also appear to influence the endothelin pathway, which matters for cardiovascular control and hypertension development. Endothelin-1 (ET-1) is the main component of this system and strongly regulates vascular tone. When people develop essential hypertension, excess ET-1 causes too much vasoconstriction, increasing vascular resistance and blood pressure (37, 38). The exercises enhance endothelial function, which then limits ET-1 production and release. Lower ET-1 activity relaxes the blood vessels, reduces peripheral resistance, and helps reduce blood pressure (35). According to the SUCRA probability ranking results, Tai Chi (82.2%) and Baduanjin (58.1%) have the highest SUCRA probabilities in reducing serum endothelin levels, indicating that the improvement effects of these two methods may be relatively more significant.

Tai Chi and Baduanjin also showed trends toward higher NO levels, though these changes were not statistically significant. This likely stems from several methodological limitations. The small sample size (*n* = 236) presents a major issue for assessing endothelial function, limiting our statistical power. The problem compounds when we consider the substantial differences across studies in training frequency, duration, and intensity, which reduce the reliability of pooled estimates. NO measurement presents additional challenges. Given the rapid degradation of NO within seconds, single serum measurements merely provide a snapshot and fail to adequately reflect the dynamic nature of endothelial function. Blood collection timing and laboratory techniques further increase variability. Future work should include larger participant groups and employ direct functional tests such as flow-mediated vasodilation to more accurately evaluate the effects of these exercises on endothelial health.

### 4.2 Differential effects of different traditional Chinese exercises

#### 4.2.1 These four traditional Chinese exercises share a common physiological mechanism, but their effects on specific cardiovascular indicators vary

Through SUCRA probability ranking analysis, these differences were quantified, revealing different effect characteristics: Tai Chi had a higher probability of being the optimal intervention in terms of blood pressure control (SBP: 86.7%, DBP: 79.3%), reduction of triglycerides (92.4%), and increase of high-density lipoprotein cholesterol (81.3%); Baduanjin ranked highest in terms of total cholesterol reduction (96.5%) and increase of nitric oxide (54.8%); Qigong guiding exercises had a relatively prominent ranking probability in blood pressure control (SBP: 81.4%, DBP: 70.1%); while Wuqinxi and Yijinjing guiding exercises had relatively lower probability rankings in other indicators. These differentiated effect rankings may be partly due to the different technical movement arrangements, breathing coordination, and physical and mental interaction techniques emphasized in different traditional exercises. In summary, this study suggests that Tai Chi and Baduanjin show higher potential advantages in improving multiple cardiovascular and metabolic indicators in the elderly, and can be prioritized as intervention choices; while Yijinjing guiding exercises also show good potential in blood pressure control. These results provide probabilistic evidence and differentiated selection references for the formulation of exercise prescriptions for specific health outcomes in the future.Technical characteristics and fitness functions of Tai Chi.

The core of Tai Chi lies in cultivating overall body capacity, and it is a practice with high demands on human motor functional capabilities (39). Tai Chi training is a continuous process of seeking integrated strength and optimizing function (40). Regarding Tai Chi's technical principles and fitness functions, the movements exhibits slow, flowing characteristics, emphasizing continuous weight shifting, semi-squat postures (high/low stances), and smooth, circular movements, requiring greater participation of lower limb and core muscle groups. This represents a form of low-intensity endurance and balance training. During movement transitions and footwork, individual techniques such as kicking, low stance, and single-leg standing require practitioners to possess considerable strength, core function, balance qualities, and flexibility.

The effects of Tai Chi on the cardiovascular system occur through improvements in cardiopulmonary function, enhanced myocardial contractility, and regulation of body balance, with continuous spatial transformations of the torso and shifts in hand and foot positions, and postural adjustments that can also remodel neuronal cells in brain cortical functional areas (41). This continuous dynamic loading and whole-body coordinated movement pattern give Tai Chi unique advantages in activating the vascular shear stress mechanism, which also explains its superior performance in blood pressure control and triglyceride reduction.

#### 4.2.2 Technical characteristics and fitness functions of baduanjin

Baduanjin is based on traditional Chinese medicine meridian and acupoint theory, with rich connotations and representative significance, as meridians are pathways that circulate qi and blood throughout the body, connecting viscera and limbs, and communicating upper and lower, internal and external (42). Baduanjin combines movement and stillness, balancing tension and relaxation, demonstrating left-right symmetry and front-back balance, which promotes coordinated bodily development with symmetrical practice enabling body balance, achieving “balanced yin and yang, lubricating joints” (43). The movements are predominantly static, emphasizing sustained postures that involve stretching and stimulating meridian points. Key technical characteristics encompass ascending and descending stances, small-range footwork, and motions such as reaching for the feet and lifting overhead, all performed while maintaining even breathing, mental relaxation, and a focus on qi.

The static stretching and sustained postures characteristic of Baduanjin likely underpin its benefits to peripheral blood flow and metabolic efficiency, offering a plausible physiological explanation for its observed efficacy in reducing TC and improving endothelial markers. In traditional Chinese medicine, this is understood as the effect of stimulating meridian pathways; future research is needed to delineate how these traditional concepts correspond to modern physiological parameters.

#### 4.2.3 Technical characteristics and fitness functions of yijinjing

The movement technique essentials of Yijinjing involve fully extending, flexing, and rotating the body, a practice characterized by “stretching tendons” and “pulling bones” (44). Through this, it achieves traction of large and small muscle groups, whole-body fascia, and joint connective tissues (e.g., tendons, ligament, joint capsules), which in turn promotes blood circulation in active soft tissues, optimizes soft tissue nutrition and metabolism, boosts the flexibility/mobility of bones, joints, and muscles, and thus enhances overall body qualities. Yijinjing's “tendon stretching and bone pulling” characteristics gives it advantages in improving myofascial elasticity and promoting peripheral blood circulation, which may be one of the reasons for its favorable performance in blood pressure control.

#### 4.2.4 Technical characteristics and fitness functions of wuqinxi

Wuqinxi techniques imitate five animals (tiger, deer, bear, ape, and crane), adopting their typical movement advantages: the tiger's forelimb lunging, the bear's lying down and standing up, the deer's head and neck rotation, the ape's toe jumping, and the crane's wing spreading (45). On this basis, Wuqinxi applies the ancient philosophical concepts of the Five Elements theory, and has been condensed over a thousand years of social practice into a “medicinal prescription.” Tiger play can invigorate energy and enrich organ qi; deer play can strengthen the liver and kidneys and enhance spleen-stomach function; bear play can calm the spirit and strengthen the body, harmonizing liver fire; ape play can sharpen hearing and vision, lightening the brain and body; crane play can smooth the meridians and activate blood circulation (46). Wuqinxi features rich movement variations and large amplitudes, theoretically possessing comprehensive cardiovascular exercise effects. However, in this study, due to the limited number of included studies, its unique advantages have not been fully demonstrated.

#### 4.2.5 Comprehensive analysis of exercise differences

Upon analyzing the technical principles and fitness functions of four traditional Chinese exercises, it becomes evident that each focuses on different aspects of improving cardiovascular metabolism. Tai Chi's fitness functions mainly emphasize overall balance and nervous system regulation, particularly excelling in improving blood pressure, enhancing vascular elasticity, and body balance capacity, with its continuous weight shifting and lower limb loading enabling effective activation of vascular shear stress and neurovascular reflex regulatory pathways, also showing favorable effects in relieving chronic stress. Baduanjin's fitness functions focus on the targeted regulation of organ functions and the facilitation of qi and blood circulation. Static stretching combined with meridian points stimulation offers clear benefits for digestive function and helps regulate glucose and lipid levels (TC in particular), while improving endothelial function. Yijinjing's health benefits come from strengthening tendons and bones and promoting meridian flow. The practice's signature “stretching and pulling” motion, relieve spinal issues, and contributes to better blood pressure control. The practice of Wuqinxi is intended to invigorate and balance the five organ systems, promoting a condition of comprehensive harmony in the body. This approach improves cardiovascular fitness, reinforces the limbs and joints, addresses metabolic syndrome, and helps ease anxiety symptoms.

Biomechanically speaking, Tai Chi functions as a dynamic balance activity that requires ongoing movement and whole-body coordination, sharing key features with aerobic endurance exercise. Baduanjin is classified as a static stretching exercise that prioritizes posture maintenance and meridian activation, focusing on flexibility and the regulation of internal organs.

Yijinjing is a strength-traction exercise, focusing on active muscular elongation and antagonistic contraction. Wuqinxi is an imitative exercise that includes a wide variety of movement patterns. These technical characteristic differences, combined with the three major physiological mechanisms, jointly determine the differential effect spectra of different exercises on specific cardiovascular metabolic indicators. Tai Chi, due to its dynamic characteristics, effectively activates hemodynamic mechanisms and neural regulatory mechanisms, thus showing outstanding performance in blood pressure and TG control; Baduanjin, due to its meridian stimulation characteristics, effectively improves visceral circulation and endothelial function, thus demonstrating significant effects in cholesterol metabolism and NO elevation.

## 5 Conclusions and future directions

This study applied network meta-analysis to evaluate the cardiovascular and metabolic effects of four traditional Chinese exercises in hypertensive patients who were middle-aged and in older adults populations. Our analysis included 1,501 participants from 19 randomized controlled trials and showed that each of the four exercise types delivered considerable cardiovascular benefits beyond what conventional treatment offered. The SUCRA probability ranking results show that Tai Chi and Baduanjin demonstrate a relatively high probability of superiority in improving multiple cardiovascular and metabolic indicators of middle-aged and older adults patients with hypertension. Out of all the exercises, Tai Chi and Baduanjin produce the widest array of improvements in cardiovascular and metabolic markers. These findings suggest that adding structured Tai Chi or Baduanjin programs to standard care could benefit middle-aged and older adults patients with hypertension and cardiovascular disease.

Our study had some limitations. First, the limited number of existing studies resulted in insufficient sample sizes and potential publication bias, a factor that may undermine the robustness of the findings. Second, raw data that was either incomplete or non-standardized made it unfeasible to adjust for variables like intervention frequency and duration in subgroup analyses. Thirdly, although the Cochrane risk of bias assessment tool was used in the study to conduct a systematic review of all the included randomized controlled trials, the assessment results showed that some studies had “high risk” or “incomplete information” in key methodological areas such as random sequence generation, allocation concealment, or blinding implementation. This heterogeneity in study quality may potentially have an impact on the accuracy of the combined effect estimation. Therefore, the SUCRA ranking probabilities presented in the research results can be regarded as the best estimate based on the currently available but unevenly quality-assessed evidence. Finally, significant heterogeneity was observed across the included studies, largely arising from variations in intervention protocols and patient baseline characteristics. These differences may consequently affect the reliability and generalizability of the findings.

Additionally, we recommend that future research focus on the following: (1) In research design, conducting multi-center, large-sample, rigorously blinded randomized controlled trials to directly compare the differential effects of different exercises (such as Tai Chi and Baduanjin), and standardizing intervention protocols (unified lineage/style, training dosage, quality control); (2) In mechanism exploration, incorporating more comprehensive biomarker systems, including hemodynamic indicators (cardiac output, peripheral resistance), endothelial function markers (flow-mediated vasodilation, circulating endothelial cells), inflammatory factors (IL-6, CRP), and autonomic nervous function (heart rate variability, baroreflex sensitivity), to elucidate the action pathways of different exercises; (3) In dose-response relationships, systematically investigating the dose-effect relationship between exercise “prescription elements”(frequency, intensity, time, type) and cardiovascular benefits to provide evidence for individualized exercise prescription development; (4) In long-term effects and adherence, extending follow-up periods (≥1 year) to evaluate long-term cardiovascular event incidence, exercise adherence and influencing factors, and exploring the feasibility of intervention models such as remote guidance and community promotion.

## Data Availability

The datasets presented in this study can be found in online repositories. The names of the repository/repositories and accession number(s) can be found in the article/Supplementary Material.
